# Trends in mortality rates for musculoskeletal and connective tissue diseases

**DOI:** 10.1097/MD.0000000000048058

**Published:** 2026-03-20

**Authors:** Chengzhuan Luo, Junsheng Jiang

**Affiliations:** a Department of Hand Surgery, Linping Branch, the Second Affiliated Hospital of Zhejiang University, Hangzhou, China; b Department of Pediatrics, Linping Branch, the Second Affiliated Hospital of Zhejiang University, Hangzhou, China.

**Keywords:** age-adjusted mortality rates, CDC, connective tissue, musculoskeletal system

## Abstract

The burden of the musculoskeletal system and connective tissue poses a significant public health challenge in the United States. Musculoskeletal diseases and connective tissue diseases independently contribute to high morbidity and mortality rates. This study seeks to investigate mortality trends associated with the simultaneous occurrence of the musculoskeletal system and connective tissue among the US population over the period from 1999 to 2020. Centers for Disease Control and Prevention’s Wide-Ranging Online Data for Epidemiologic Research was used to identify the musculoskeletal system and connective tissue-related deaths that occurred within the United States from 1999 to 2020. Crude and age-adjusted mortality rates (AAMRs) were calculated, as well as annual percent change and weighted average annual percent change with 95% confidence intervals for the AAMRs. The Joinpoint Regression Program was used to determine trends in mortality within the study period. Joinpoint regression analysis was employed to determine annual percentage changes and assess statistical significance (*P* < .05). From 1999 to 2020, female patients demonstrated greater mortality rates from the musculoskeletal system and connective tissue compared with males. When stratified by race and ethnicity, Black patients had the highest AAMR over the study period at 9.50 per 100,000 people in 1999. Additionally, AAMRs were consistently higher in rural areas compared with urban locations. By age group, patients aged 85+ years had the highest overall crude mortality rate at 91.20 per 100,000 people in 1999, with the lowest rate in ages 25 to 34 years at 0.70 per 100,000 people in 1999. This study expands upon previously reported trends in musculoskeletal system and connective tissue-related mortality, highlighting epidemiological differences in musculoskeletal system and connective tissue-related death. Significant disparities in mortality rates were noted in older-aged, female, Black, and rural patients. Targeted public health strategies concerning the unique needs of these diverse populations will be essential in improving mortality rates.

## 1. Introduction

Musculoskeletal system and connective tissue diseases (MSCTDs) represent a diverse group of disorders that affect the bones, joints, muscles, and connective tissues, imposing a substantial burden on public health systems worldwide.^[1,2]^ In the United States, these conditions not only lead to chronic pain, functional impairment, and reduced quality of life for millions of individuals but also contribute significantly to morbidity and mortality, creating long-term challenges for healthcare delivery and resource allocation.^[3]^ With an aging population, shifting demographic patterns, and evolving environmental and lifestyle factors, understanding the mortality trends associated with MSCTDs has become increasingly critical for guiding public health policies and targeted interventions.

Prior epidemiological studies have documented the prevalence and impact of individual MSCTDs, such as rheumatoid arthritis, osteoporosis, and systemic lupus erythematosus, but few have comprehensively analyzed the overall mortality trends of these diseases as a collective group over an extended period.^[4,5]^ Existing research has highlighted disparities in disease outcomes across different demographic groups, with factors such as age, sex, race/ethnicity, and geographic location emerging as potential influencers of mortality risk. For instance, older adults have been shown to face higher mortality rates due to age-related declines in physiological function and comorbidities, while racial and ethnic minorities often experience barriers to accessing quality healthcare, which may exacerbate disease progression and mortality.^[6]^ However, gaps remain in understanding how these disparities have evolved over time, particularly in the context of changing healthcare practices and public health initiatives implemented over the past 2 decades.

The Centers for Disease Control and Prevention’s Wide-Ranging Online Data for Epidemiologic Research (CDC-WONDER) database provides a robust source of population-level mortality data, enabling researchers to examine trends in disease-related deaths across diverse demographic and geographic groups.^[7]^ Understanding the mortality patterns of MSCTDs across different populations is essential for developing effective public health strategies. This study not only expands upon previous research by providing a comprehensive analysis of MSCTD-related mortality over a 22-year period but also offers valuable insights that can inform policy decisions and healthcare practices to improve outcomes for individuals affected by these debilitating conditions.

## 2. Methods

### 2.1. Data source

Mortality data related to MSCTDs were extracted from the CDC-WONDER database. This database aggregates comprehensive population-level mortality records from death certificates across the United States, including detailed information on the demographic characteristics of decedents and the underlying cause of death. The study period covered was from 1999 to 2020, ensuring a long-term perspective to capture temporal trends in MSCTD-related mortality. As this study employed deidentified public data made available by the government, it was not necessary to obtain formal approval from the regional institutional review board. However, the study adhered to all relevant laws, regulations, and Strengthening the Reporting of Observational Studies in Epidemiology guidelines for observational studies, ensuring adherence to the highest research ethics standards.

### 2.2. Study population and case definition

The study population included all individuals who died in the United States during the study period with an underlying cause of death classified as a musculoskeletal system or connective tissue disease. The classification was based on the International Classification of Diseases (ICD) codes, with ICD-10 codes used for deaths occurring from 1999 onward. Cases were identified using relevant ICD-10 codes ranging from M00 to M19 corresponding to disorders of the musculoskeletal system and connective tissue, ensuring consistency and accuracy in case ascertainment.

### 2.3. Variables

The data extraction also included demographic and regional information, which encompassed variables such as gender, racial/ethnic background, age, urban-rural classification, census region, and state of residence. In terms of urban-rural classifications, we employed the National Center for Health Statistics Urban-Rural Classification Scheme, which is based on the 2013 US census categorization. This scheme differentiates counties into urban areas (which include large metropolitan areas with populations of 1 million or more, as well as medium and small metropolitan areas with populations ranging from 50,000 to 999,999) and rural areas (defined as those with populations below 50,000). The National Center for Health Statistics 2013 classification has been consistently applied. The census regions were organized into the Northeast, Midwest, South, and West, adhering to the definitions provided by the US Census Bureau.

### 2.4. Statistical analysis

From the extracted data, musculoskeletal system and connective tissue-related crude and age-adjusted mortality rates (AAMRs) in patients were calculated. We standardized the AAMR per 100,000 persons using the 2000 US standard population, as previously described. In order to determine trends in mortality within the study period, we used the Joinpoint Regression Program (Joinpoint version 4.9.0.0 available from National Cancer Institute, Bethesda). The annual percentage change (APC) and average annual percent change (AAPC) with 95% confidence intervals (CIs) for the respective AAMRs were calculated using the Monte Carlo permutation test, which used the line segments to link a join point. Using a statistical significance of *P* ≤ .05, APC and AAPCs were determined to be increasing or decreasing if the slope describing the change in mortality throughout the time interval was significantly different from 0 using a 2-tailed *t* test. Throughout the data analysis and results, asterisks were used to denote significance.

## 3. Results

### 3.1. Overall mortality trends

**Figure 1.**
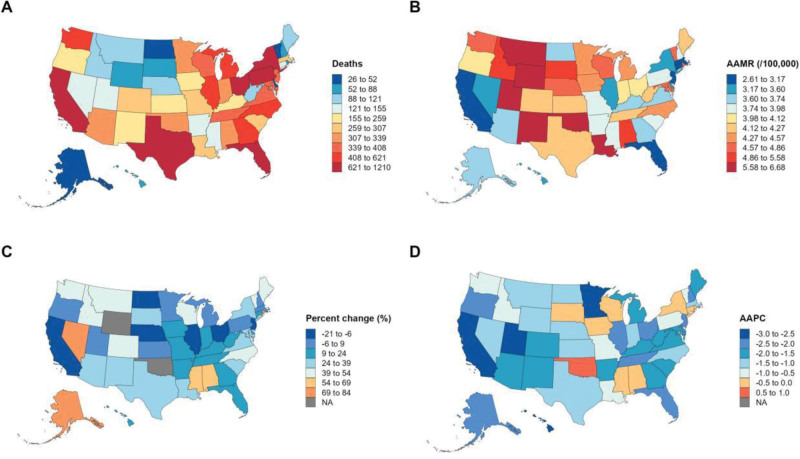
Overall mortality trends. AAMR = age-adjusted mortality rate, AAPC = average annual percent change.

**Figure 2.**
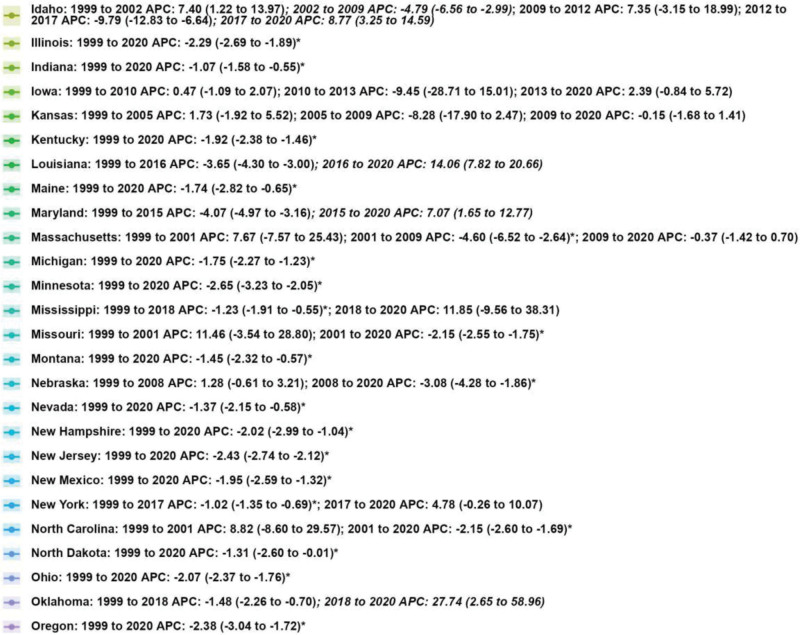
Mortality by region. APC = annual percentage change.

From 1999 to 2020, a total of 13,144 deaths related to MSCTDs were recorded in the United States in 1999, increasing to 15,271 in 2020, representing a 16.18% overall increase in the number of deaths. By contrast, the AAMR showed a significant downward trend over the same period. The AAMR was 7.40 per 100,000 population (95% CI: 7.30–7.60) in 1999 and decreased to 5.80 per 100,000 population (95% CI: 5.70–5.90) in 2020, with an AAPC of −1.22% (95% CI: −1.75 to −0.69, *P* < .05; Table 1, Figs. 1 and 2).

**Table 1:** Musculoskeletal system and connective tissue disease deaths and AAMR in the United States from 1999 to 2020 and their changing trends.

Characteristic	Deaths			AAMR		
	1999	2020	Percent change (%)	1999 (95% CI)	2020 (95% CI)	AAPC (95% CI)
Total	13,144	15,271	16.18	7.40 (7.30–7.60)	5.80 (5.70–5.90)	−1.22 (−1.75 to −0.69)*
Sex						
Female	9393	9161	−2.47	8.90 (8.70–9.10)	6.20 (6.10–6.40)	−1.71 (−2.25 to −1.17)*
Male	3751	6110	62.89	5.40 (5.20–5.60)	5.20 (5.10–5.40)	−0.22 (−0.87 to 0.44)
Census region						
Northeast	2295	2536	10.50	6.20 (5.90–6.40)	5.20 (4.90–5.40)	−0.90 (−1.71 to −0.08)*
Midwest	3445	3596	4.38	8.10 (7.90–8.40)	6.30 (6.10–6.60)	−1.29 (−1.90 to −0.67)*
South	4573	5975	30.66	7.40 (7.20–7.60)	6.00 (5.90–6.20)	−1.10 (−1.87 to −0.32)*
West	2831	3164	11.76	8.10 (7.80–8.40)	5.30 (5.10–5.50)	−2.50 (−2.74 to −2.26)*
Race						
Hispanic	676	1208	78.70	6.80 (6.20–7.30)	4.30 (4.10–4.60)	−2.02 (−2.62 to −1.43)*
NH Black	1590	2327	46.35	9.50 (9.10–10.00)	8.80 (8.40–9.10)	−0.54 (−1.47 to 0.40)
NH White	10,567	11,172	5.73	7.10 (7.00–7.30)	5.60 (5.50–5.70)	−1.15 (−1.77 to −0.52)*
NH Other	268	544	102.99	5.10 (4.50–5.80)	3.50 (3.20–3.80)	−2.31 (−2.79 to −1.84)*
Urbanization						
Metropolitan	10,577	12,188	15.23	7.40 (7.20–7.50)	5.50 (5.40–5.60)	−1.54 (−1.95 to −1.13)*
Nonmetropolitan	2567	3083	20.10	7.70 (7.40–8.00)	7.30 (7.00–7.60)	−0.29 (−1.26 to 0.69)
Age groups†						
25–34 yr	291	248	−14.78	0.70 (0.60–0.80)	0.50 (0.50–0.60)	−1.50 (−2.16 to −0.84)*
35–44 yr	636	524	−17.61	1.40 (1.30–1.50)	1.20 (1.10–1.40)	−0.98 (−2.09 to 0.14)
45–54 yr	1004	1007	0.30	2.70 (2.60–2.90)	2.50 (2.30–2.60)	−0.63 (−1.68 to 0.43)
55–64 yr	1284	2154	67.76	5.40 (5.10–5.70)	5.10 (4.90–5.30)	−0.21 (−0.73 to 0.31)
65–74 yr	2342	3372	43.98	12.70 (12.20–13.20)	10.40 (10.00–10.70)	−1.07 (−1.55 to −0.59)*
75–84 yr	3797	3773	−0.63	31.10 (30.10–32.00)	22.90 (22.20–23.70)	−1.40 (−2.46 to −0.33)*
85+ yr	3790	4193	10.63	91.20 (88.30–94.10)	63.00 (61.10–64.90)	−1.82 (−2.43 to −1.20)*

AAMR = age-adjusted mortality rate, AAPC = average annual percent change, CI = confidence interval, NH = non-Hispanic.

† For the age groups, the crude mortality rate was used as a substitute for AAMR, and the AAPC was computed based on the crude mortality rate.

* *P* < .05.

### 3.2. Mortality by sex

**Figure 3.**
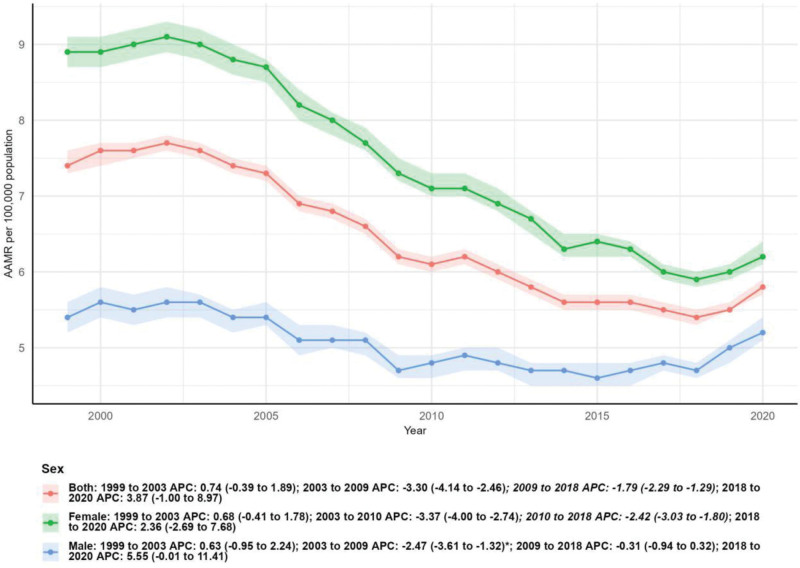
Mortality by sex. AAMR = age-adjusted mortality rate, APC = annual percentage change.

Distinct gender differences were observed in MSCTD-related mortality trends. The number of female deaths decreased slightly from 9393 in 1999 to 9161 in 2020 (−2.47%), while the AAMR declined significantly from 8.90 per 100,000 (95% CI: 8.70–9.10) to 6.20 per 100,000 (95% CI: 6.10–6.40), with an AAPC of −1.71% (95% CI: −2.25 to −1.17, *P* < .05). By contrast, the number of male deaths increased substantially by 62.89%, from 3751 in 1999 to 6110 in 2020. However, the male AAMR remained relatively stable, decreasing slightly from 5.40 per 100,000 (95% CI: 5.20–5.60) to 5.20 per 100,000 (95% CI: 5.10–5.40), with a nonsignificant AAPC of −0.22% (95% CI: −0.87 to 0.44, *P* > .05). Joinpoint regression analysis further revealed that the overall mortality trend for both sexes combined showed 3 distinct phases: a nonsignificant increase from 1999 to 2003 (APC: 0.74%, 95% CI: −0.39 to 1.89), a significant decline from 2003 to 2009 (APC: −3.30%, 95% CI: −4.14 to −2.46, *P* < .05), and a continued significant decrease from 2009 to 2018 (APC: −1.79%, 95% CI: −2.29 to −1.29, *P* < .05), followed by a nonsignificant increase from 2018 to 2020 (APC: 3.87%, 95% CI: −1.00 to 8.97; Table 1 and Fig. 3).

### 3.3. Mortality by census region

**Figure 4.**
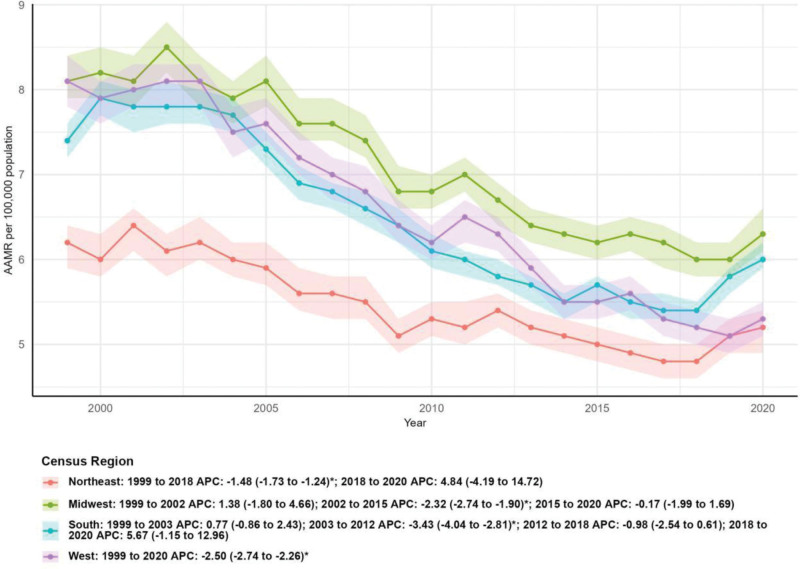
Mortality by census region. AAMR = age-adjusted mortality rate, APC = annual percentage change.

All 4 US census regions exhibited a decrease in AAMR over the study period, although the magnitude of the decline varied. The West region showed the most pronounced reduction, with an AAPC of −2.50% (95% CI: −2.74 to −2.26, *P* < .05), followed by the Midwest (AAPC: −1.29%, 95% CI: −1.90 to −0.67, *P* < .05), the South (AAPC: −1.10%, 95% CI: −1.87 to −0.32, *P* < .05), and the Northeast (AAPC: −0.90%, 95% CI: −1.71 to −0.08, *P* < .05). The number of deaths increased across all regions, with the largest percentage increase in the South (30.66%), followed by the West (11.76%), the Northeast (10.50%), and the Midwest (4.38%). Joinpoint analysis indicated that the West region maintained a consistent downward trend throughout 1999 to 2020, while the Northeast, Midwest, and South showed phase-specific fluctuations, including a nonsignificant increase in the Northeast and South during 2018 to 2020 (Table 1 and Figs. 1, 2, and 4).

### 3.4. Mortality by race/ethnicity

**Figure 5.**
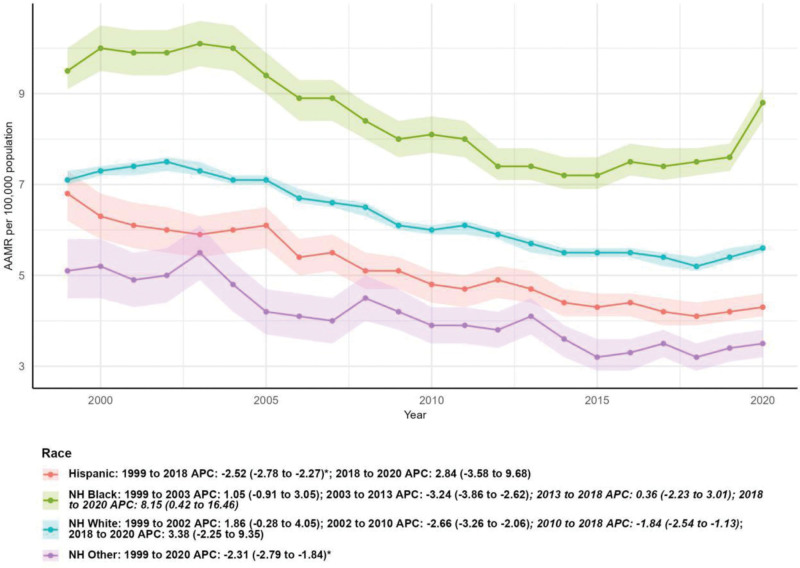
Mortality by race/ethnicity. AAMR = age-adjusted mortality rate, APC = annual percentage change, NH = non-Hispanic.

Significant variations in mortality trends were observed across racial/ethnic groups. The number of deaths increased most dramatically among non-Hispanic (NH) Other individuals (102.99%), followed by Hispanic (78.70%), NH Black (46.35%), and NH White (5.73%) populations. However, AAMR decreased significantly for all groups except NH Black individuals. The AAPC was −2.02% (95% CI: −2.62 to −1.43, *P* < .05) for Hispanic individuals, −1.15% (95% CI: −1.77 to −0.52, *P* < .05) for NH White individuals, and −2.31% (95% CI: −2.79 to −1.84, *P* < .05) for NH Other individuals. The NH Black population had the highest AAMR in both 1999 (9.50 per 100,000, 95% CI: 9.10–10.00) and 2020 (8.80 per 100,000, 95% CI: 8.40–9.10), with a nonsignificant AAPC of −0.54% (95% CI: −1.47 to 0.40, *P* > .05). Joinpoint analysis revealed that NH Black individuals experienced a significant decline in AAMR from 2003 to 2013 (APC: −3.24%, 95% CI: −3.86 to −2.62, *P* < .05) but a significant increase from 2018 to 2020 (APC: 8.15%, 95% CI: 0.42–16.46, *P* < .05; Table 1 and Fig. 5).

### 3.5. Mortality by urbanization level

**Figure 6.**
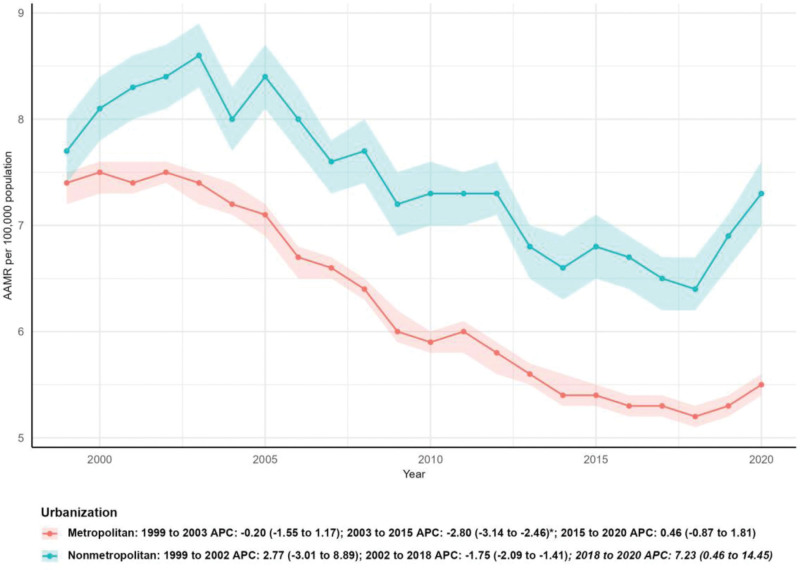
Mortality by urbanization level. AAMR = age-adjusted mortality rate, APC = annual percentage change.

Metropolitan areas had a larger number of deaths (10,577 in 1999 vs 12,188 in 2020, 15.23% increase) compared with nonmetropolitan areas (2567 in 1999 vs 3083 in 2020, 20.10% increase). The AAMR in metropolitan areas decreased significantly from 7.40 per 100,000 (95% CI: 7.20–7.50) to 5.50 per 100,000 (95% CI: 5.40–5.60), with an AAPC of −1.54% (95% CI: −1.95 to −1.13, *P* < .05). By contrast, nonmetropolitan areas showed a nonsignificant decline in AAMR (from 7.70 per 100,000, 95% CI: 7.40–8.00 to 7.30 per 100,000, 95% CI: 7.00–7.60), with an AAPC of −0.29% (95% CI: −1.26 to 0.69, *P* > .05). Joinpoint analysis indicated that nonmetropolitan areas experienced a significant increase in mortality from 2018 to 2020 (APC: 7.23%, 95% CI: 0.46–14.45, *P* < .05), while metropolitan areas showed a stable downward trend until 2015, followed by a nonsignificant increase (Table 1 and Fig. 6).

### 3.6. Mortality by age group

**Figure 7.**
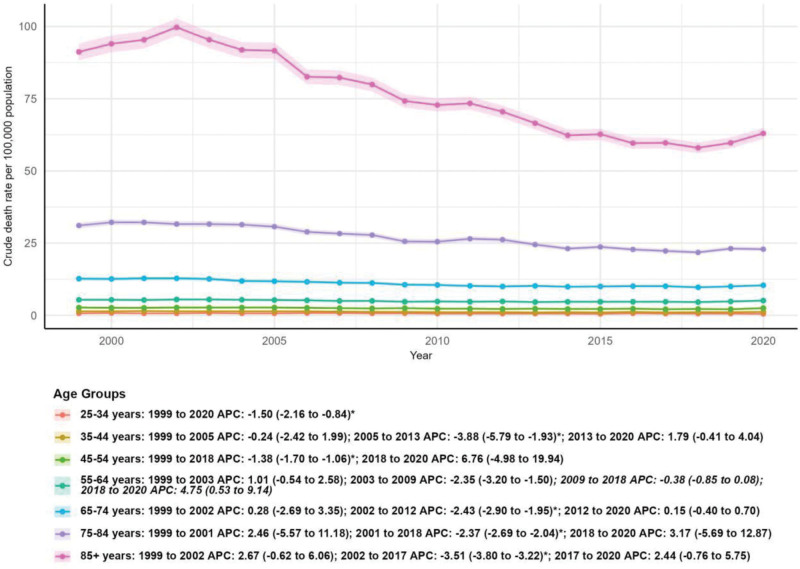
Mortality by age group. APC = annual percentage change.

The oldest age group (85+ years) had the highest crude mortality rate throughout the study period, with 3790 deaths in 1999 (91.20 per 100,000) and 4193 deaths in 2020 (63.00 per 100,000), showing a significant downward trend (AAPC: −1.82%, 95% CI: −2.43 to −1.20, *P* < .05). The 75 to 84 years age group also exhibited a significant decline in crude mortality rate (AAPC: −1.40%, 95% CI: −2.46 to −0.33, *P* < .05). By contrast, the number of deaths increased substantially in the 55 to 64 years (67.76%) and 65 to 74 years (43.98%) age groups, although their crude mortality rates remained relatively stable. The youngest age groups (25–34 years and 35–44 years) had the lowest mortality rates, with the 25 to 34 years age group showing a significant decline (AAPC: −1.50%, 95% CI: −2.16 to −0.84, *P* < .05) and the 35 to 44 years age group showing a nonsignificant decrease (AAPC: −0.98%, 95% CI: −2.09 to 0.14, *P* > .05). Joinpoint analysis revealed phase-specific trends for most age groups, including a significant increase in the 55 to 64 years age group during 2018 to 2020 (APC: 4.75%, 95% CI: 0.53 to 9.14, *P* < .05; Table 1 and Fig. 7).

## 4. Discussion

This study comprehensively analyzed the mortality trends of MSCTDs in the United States from 1999 to 2020 using data from the CDC-WONDER database, revealing complex patterns stratified by demographic, geographic, and socioeconomic factors. The key findings highlight a paradoxical trend: while the total number of MSCTD-related deaths increased by 16.18%, the AAMR declined significantly by an average of 1.22% annually. This discrepancy may be primarily attributed to the aging of the US population, as older adults are disproportionately affected by MSCTDs and account for a growing share of the total population.^[8]^ Additionally, improvements in disease diagnosis and reporting systems over the past 2 decades may have enhanced the identification of MSCTD-related deaths, contributing to the increase in absolute numbers, while standardized rates reflect true reductions in mortality risk.

The distinct gender-specific trends observed in this study align with previous research indicating higher susceptibility to certain MSCTDs among women, such as rheumatoid arthritis and systemic lupus erythematosus. Studies have confirmed that estrogen is closely related to the occurrence of these diseases among many pathogenic factors.^[9]^ The significant decline in female AAMR may reflect advancements in targeted therapies, improved access to rheumatological care, and enhanced disease management strategies for women. By contrast, the substantial increase in male deaths without a corresponding significant change in AAMR suggests that the male population affected by MSCTDs is expanding, potentially due to changing lifestyle factors (e.g., increased obesity and sedentary behavior) or underdiagnosis in earlier years.^[10]^ The stable male AAMR also raises concerns about potential disparities in healthcare access or treatment adherence among men, highlighting the need for gender-specific public health interventions to address unmet needs in this population.

All 4 US census regions demonstrated significant declines in AAMR, with the West region showing the most dramatic reduction. This may be associated with variations in healthcare infrastructure, access to specialized care, and public health initiatives across regions. The South, which had the largest percentage increase in total deaths, continues to face challenges related to health disparities, including higher rates of poverty, limited access to healthcare in rural areas, and higher prevalence of comorbidities (e.g., diabetes and hypertension) that may exacerbate MSCTD outcomes.^[11,12]^

Urban-rural disparities were also prominent, with nonmetropolitan areas showing a nonsignificant decline in AAMR compared with the significant reduction in metropolitan areas. The significant increase in mortality in nonmetropolitan areas during 2018 to 2020 may be linked to the COVID-19 pandemic, which disproportionately disrupted healthcare access in rural communities, or long-standing systemic issues such as shortages of healthcare providers, limited access to specialty care, and lower health literacy.^[13–15]^ These findings underscore the need to strengthen healthcare delivery systems in rural and underserved regions to reduce persistent mortality disparities.

Racial and ethnic differences in MSCTD mortality highlight ongoing health inequities in the United States. NH Black individuals maintained the highest AAMR throughout the study period, with a nonsignificant annual decline, in contrast to significant reductions among Hispanic, NH White, and NH Other populations. This aligns with broader health disparities observed across multiple disease categories, driven by factors such as systemic racism, limited access to quality healthcare, socioeconomic disadvantage, and higher rates of comorbidities.^[16–18]^ The significant increase in AAMR among NH Black individuals during 2018 to 2020 is particularly concerning and may reflect the disproportionate impact of the COVID-19 pandemic on marginalized communities, as well as gaps in pandemic-related healthcare resources and disease management.^[19]^

The substantial increase in the number of deaths among NH Other and Hispanic populations may be attributed to population growth and improved diagnosis, while their significant AAMR declines suggest that targeted interventions and improved healthcare access for these groups are yielding positive outcomes. However, continued efforts are needed to address structural barriers to healthcare and reduce racial/ethnic disparities in MSCTD mortality.^[20]^

The oldest age groups (75–84 years and 85+ years) exhibited the highest mortality rates but also significant declines in crude mortality rates, reflecting advancements in geriatric care, improved disease management, and better control of comorbidities. The significant increase in deaths among the 55 to 64 years and 65 to 74 years age groups (67.76% and 43.98%, respectively) may be a result of the aging baby boomer generation entering these age brackets, as well as potential delays in diagnosis or treatment in middle-aged adults. The stable crude mortality rates in these groups suggest that current interventions are not fully addressing the growing burden, highlighting the need for early detection and targeted disease management strategies for middle-aged populations.^[21,22]^

The youngest age groups (25–34 years and 35–44 years) had the lowest mortality rates, with a significant decline in the 25 to 34 years age group. This may be due to the lower prevalence of severe MSCTDs in younger adults and improved access to care for this demographic. However, the nonsignificant decline in the 35 to 44 years age group warrants attention, as early-onset MSCTDs can have long-term impacts on quality of life and may contribute to increased mortality in later years if not properly managed.^[23–25]^

### 4.1. Strengths and limitations

A key strength of this study is its use of population-level data from the CDC-WONDER database, which provides comprehensive and reliable mortality records across the United States over a 22-year period. The stratified analysis by age, sex, race/ethnicity, census region, and urbanization level allowed for the identification of specific disparities and trends that might otherwise be overlooked. Additionally, the use of joinpoint regression analysis enabled the detection of phase-specific changes in mortality trends, providing insights into temporal shifts in MSCTD outcomes.

However, this study has several limitations. First, the reliance on death certificates for case identification may be subject to misclassification bias, as the underlying cause of death may be inaccurately reported, particularly for complex diseases with multiple comorbidities. Second, the study did not account for potential confounders, such as socioeconomic status, access to healthcare, lifestyle factors, or specific MSCTD subtypes, which may influence mortality outcomes. Third, the use of crude mortality rates for age groups, while necessary due to inherent age stratification, limits comparisons across different age brackets. Future studies should incorporate additional variables and subtype-specific analyses to further elucidate the factors driving MSCTD mortality trends.

## 5. Conclusion

The findings of this study have important implications for public health policy and clinical practice. Targeted interventions are needed to address disparities in MSCTD mortality among older adults, NH Black individuals, rural populations, and men. This may include expanding access to specialized rheumatological care in underserved regions, implementing culturally tailored health education programs, improving disease screening and early detection, and addressing social determinants of health, such as poverty, housing insecurity, and healthcare access.

In conclusion, this study provides a comprehensive overview of MSCTD-related mortality trends in the United States from 1999 to 2020, identifying significant disparities across demographic and geographic groups. While overall AAMR has declined, persistent inequities highlight the need for targeted public health strategies to improve outcomes for vulnerable populations. By addressing these disparities and continuing to advance disease management and healthcare access, we can reduce the burden of MSCTDs and improve mortality rates for all Americans.

## Author contributions

**Methodology:** Chengzhuan Luo.

**Formal analysis:** Chengzhuan Luo.

**Software:** Junsheng Jiang.

**Supervision:** Junsheng Jiang.

**Validation:** Junsheng Jiang.

**Writing – original draft:** Chengzhuan Luo.

**Writing – review & editing:** Junsheng Jiang.
